# Different responses of cervical intervertebral disc caused by low and high virulence bacterial infection: a comparative study in rats

**DOI:** 10.1080/21655979.2022.2075305

**Published:** 2022-05-19

**Authors:** Jie Li, Yilei Chen, Hao Wu, Zhi Shan, Dikai Bei, Kaifeng Gan, Junhui Liu, Xuyang Zhang, Binhui Chen, Jian Chen, Feng-Dong Zhao

**Affiliations:** a Department of Orthopaedic Surgery, Sir Run Run Shaw Hospital, Zhejiang University School of Medicine, Hangzhou, Zhejiang, China; b Key Laboratory of Musculoskeletal System Degeneration and Regeneration Translational Research of Zhejiang Province, Hangzhou, Zhejiang, China; c Department of Orthopaedic Surgery, Ningbo Medical Center Li Huili Hospital, Ningbo, Zhejiang, China; d Department of Orthopaedics and Traumatology, the University of Hong Kong, Hong Kong, SAR, China

**Keywords:** Cervical intervertebral discs, disc low virulent bacterial infections, discitis staphylococcus, Propionibacterium acnes

## Abstract

The aims of this study were to investigate the outcomes of low- and high-virulence bacterial cervical intervertebral discs (IVDs) infection and its association with cervical IVDs degeneration in rats. A total of 75 clean grade male rats were used to establish the corresponding animal models of low and high virulent bacterial cervical disc infection via an anterior cervical approach, with injection of Propionibacterium acnes (P. acnes) and Staphylococcus epidermidis (S. epidermidis) with a 29 G needle to cervical IVDs. Specimens were collected for evaluation of Blood routine (Blood-RT), histological staining, and gene expression assays after a magnetic resonance imaging (MRI) scan. There were no statistical differences in all groups in white blood cells (WBC) at 2 and 6 weeks postoperatively (P = 0.136). The highest percentage of neutrophils was found in the S. epidermidis group at 2 weeks postoperatively (P = 0.043). MRI and histology showed that at 6 weeks postoperatively, the puncture group and P. acnes group had similar disc degeneration. In the S. epidermidis group, the disc and subchondral bone structure had been destroyed and bony fusion had occurred after the discitis. The upregulation of pro-inflammatory factor expression had the strongest effect of S. epidermidis on the early stage, while the upregulation in the puncture and P. acnes groups was more persistent. P. acnes infection of the cervical IVDs can lead to degenerative changes, whereas S. epidermidis infection leads to the manifestation of septic discitis. The correlation between P. acnes infection and cervical IVDs degeneration found in clinical studies was confirmed.

## Graphical Abstract

**Figure:**
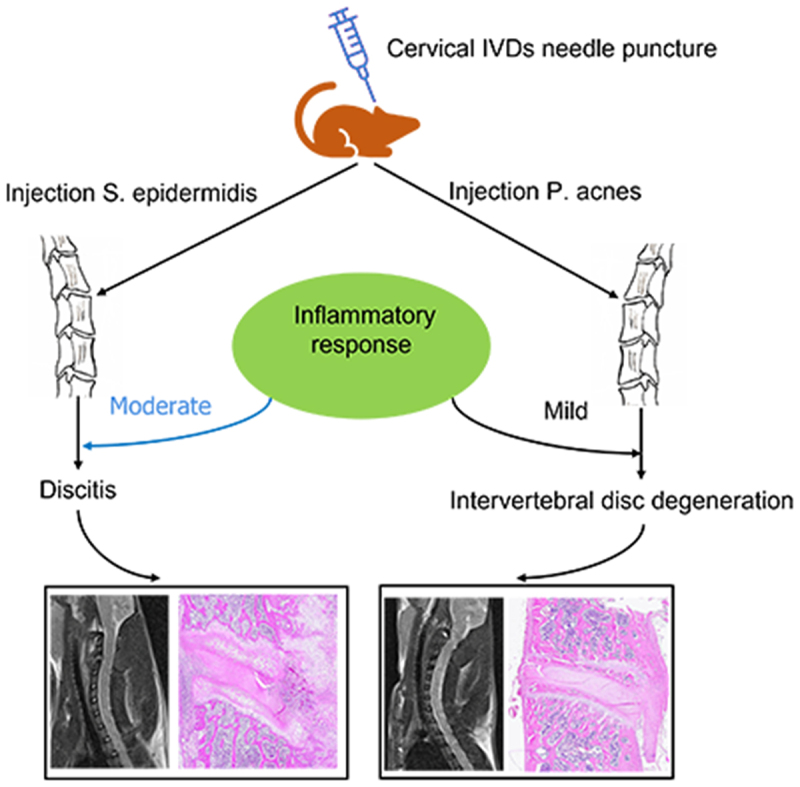

## Highlights

- P. acnes have an important role in cervical IVDs degeneration, inducing a mild inflammatory response in rats.
- S. epidermidis cervical IVDs infection led to moderate inflammation resulting in septic dis- citis.
- P. acnes mildly upregulated the low levels of the inflammatory factors TNF-α, IL-1β, in rats with IVDs, but upregulation persisted for a longer period of time than S. epidermidis.

## Introduction

Epidemiological research has found that about half of adults have experienced chronic neck pain (CNP) in their lifetime [1]. CNP is closely related to degenerative diseases of the cervical spine, and the main causes include cervical intervertebral discs (IVDs) degeneration, cervical disc herniation, cervical radiculopathy, and cervical synovial joint degeneration.

In recent years, low virulent bacterial infection has been viewed as an essential factor leading to IVDs degeneration, especially when accompanied by Modic changes in the adjacent endplates (EP) [2]. Several studies have reported the detection of bacteria in degenerated lumbar IVDs tissue. Among these bacteria, Propionibacterium acnes (P. acnes) was the most common, followed by Staphylococcus epidermidis (S. epidermidis) [3]. It has been a point of controversy as to whether they are true infections or intraoperative contamination, due to these bacteria are skin colonizing bacteria and the most common pathogens of implant-associated infections in the beginning. However, recent evidence suggested that bacteria are present in the disc before acquisition of disc tissue [4]. The detection of bacteria in degenerated cervical IVDs has been discussed in our study previously, and both bacteria were found to be predominant, but S. epidermidis was more common than P. acnes [5]. This result does not completely exclude the possibility of contamination. So far, there are no experimental studies on the pathogenicity and outcome of S. epidermidis infection of IVDs. Whether S. epidermidis infection of IVDs may be a causative factor for the clinically observed IVDs degeneration, and whether it has different effects compared with P. acnes infection of IVDs remains unclear.

Although several studies have reported experimental models of IVDs or EP degeneration induced by P. acnes injection into the lumbar or tail IVDs of animals, the results of the lumbar spine cannot simply be applied to the cervical spine, due to the anatomical structure differences [6,7]. We hypothesize that P. acnes infection of the cervical IVDs can lead to degenerative changes, and it is different from the discitis caused by S. epidermidis infection. The aim of this study is to verify whether the P. acnes, a low virulent bacterium, play any potential role in cervical IVDs degeneration and whether the response of cervical IVDs caused by two different virulent bacteria, P. acnes, and S. epidermidis, is similar. Hence, a model of cervical IVDs infection with two different virulent bacteria of SD rats was established to investigate the variations in outcomes and its association with cervical IVDs degeneration. Subsequently, the different changes of cervical IVDs degeneration-related blood analysis, MRI imaging, histological characteristics, and mRNA expression levels were evaluated. The results of this study provide a new perspective for the study of cervical IVDs degeneration.

## Materials and methods

### Culture and strain identification of P. acnes and S. epidermidis

**Figure 1.**
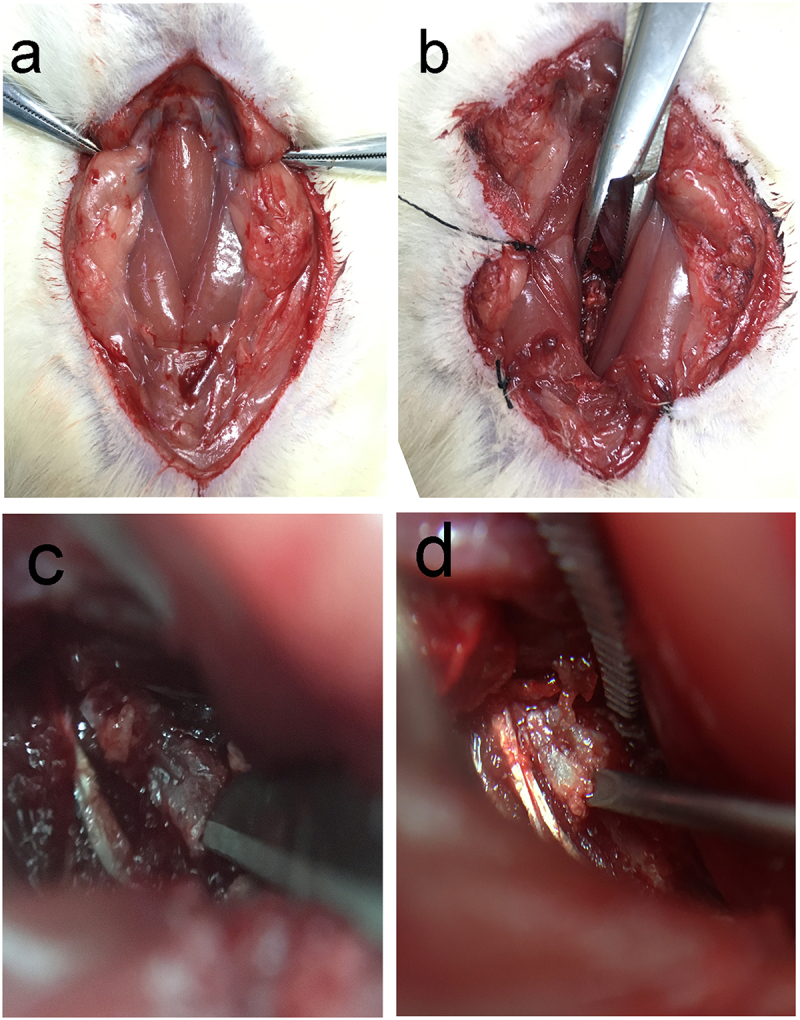
Schematic diagram of the anterior cervical spine modeling procedure, (a) Separation of the anterior cervical muscles, (b) exposure of the vertebral body and intervertebral disc, (c) disc puncture with blade, (d)disc injection.

75 clean grade male rats, with an age of approximately 8 weeks and an average body weight of 200 g, range from 190 g to 210 g, were used in this study. Animals were provided by the Zhejiang University of Traditional Chinese Medicine Laboratory Animal Center. The animals were confirmed to be free of spinal disease by preoperative imaging. All animals were operated following the ARRIVE guidelines and the U.K. Animals (Scientific Procedures) Act, 1986 and associated guidelines. After 5 days of adaptation, the rats were scanned with MRI and randomly divided into a P.acnes injection group, S. epidermidis injection group, and sharp puncture group and control group (15 in each group). Then, all rats were anesthetized with pentobarbital sodium (30 mg/kg) by intraperitoneal push, and the experimental animals were placed in the supine position and fixed on the operating table. After aseptic skin preparation, a median longitudinal incision of approximately 2.5 cm was made in the front of the neck. The anterior cervical fascia was then incised, and the sternocleidomastoid muscle was incised longitudinally in the direction of the muscle fibers to expose the trachea (Figure 1(a,b)). A blunt separation was made immediately to the right of the trachea toward the anterior cervical spine, and the tracheoesophageal sheath was pushed to the left and freed to the anterior vertebral space to expose the long cervical muscle. The cervicalis longus muscle is pulled away to the sides to reveal the C3-C6 vertebral body and fully expose the IVDs [8]. The C3-C6 IVDs were selected to complete the corresponding operations in each group. The nucleus pulposus was punctured using a fine 29 G needle in an anterior-inferior to the posterior direction along the direction of the cervical disc (Figure 1(c,d)). The whole operation was strictly aseptic and no prophylactic antibiotics were used.

**Figure 2.**
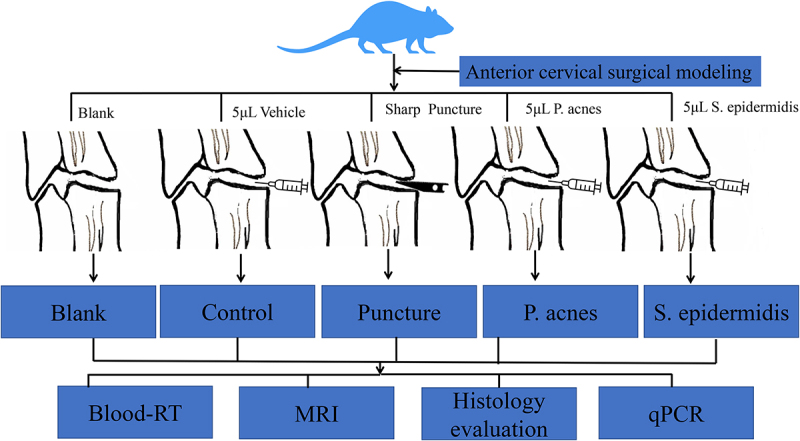
Overall flow chart of the study.

In the blank group animals, all cervical IVDs did not undergo any interventions. In the control animals, one of the IVDs were injected with 5 μl normal saline (‘Vehicle’). In rats underwent sharp puncture, the annulus fibrosus of one of the C3-C6 IVDs were punctured by a number 11 blade. In rats with P. acnes injection, 5 μl P. acnes (ATCC^#^6919 provided by Guangzhou Type Culture Collection, 1.6 × 10^8^ CFU/mL supported with NS) was injected into the one of the C3-C6 IVDs. In rats with S. epidermidis injection, 5 μl S. epidermidis (CMCC(B)^#^26069 provided by Guangzhou Type Culture Collection, 1.6 × 10^8^ CFU/mL supported with NS) was injected into one of the C3-C6 IVDs. Since the syringe needle may cause some damage to the cervical intervertebral disc, the control group distinguished the damage to the intervertebral space caused by the needle from the specific effects of bacterial infection. The overall study design flow chart is as follows (Figure 2).

### Animal complications condition and weight assessment

Intraoperative vascular and spinal cord injuries and the postoperative complications such as incisional bleeding, ventricular rest, infection, limb paralysis, and eating disorders were evaluated. Weight changes at 2, 4, and 6 weeks postoperatively were recorded.

### Blood leukocyte count and neutrophil percentage

Before the animals were executed at 2 weeks and 6 weeks postoperatively using an overdose of sodium pentobarbital, 2 ml of blood was taken from the heart into EDTA anticoagulation tubes, stored temporarily at 4°C, and the samples were sent for routine blood analysis as soon as possible.

### MR imaging and evaluation

**Figure 3.**
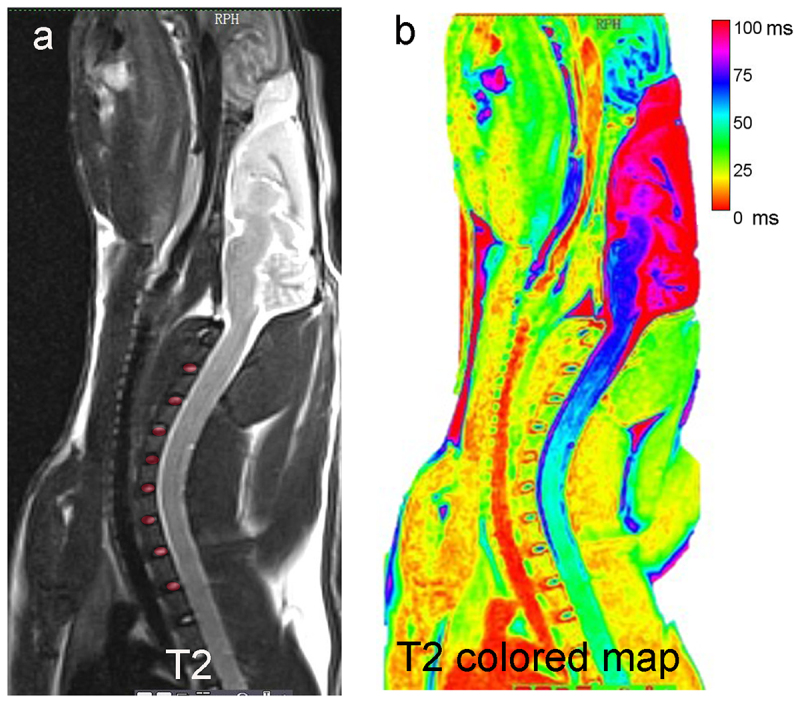
Schematic diagram of T2 value and disc volume MRI measurements. (a) ROI was selected for the IVDs in T2WI, disc volume = the area of ROI multiplies by the number of layers of the MRI scan, (b) disc and endplate T2 relaxation times were measured in T2 colored map.

Cervical spine MRI was performed at 2 weeks, and 6 weeks postoperatively, with a GE Sigma CV/I (3.0 T) using an animal surface coil to observe signal changes of cervical intervertebral disc on T2-weighted images and T2 colored map [9]. Cervical IVDs and EP T2 value and disc volume were measured (Figure 3(a,b)). The parameters are as follows: scanning conditions for T2-weighted images in the sagittal plane were repetition/echo times of 2500/120 ms, the bandwidth of 31.25 Hz/Px, and echo train length of 17. The scanning conditions for T1-weighted were repetition/echo times mini full of 550 ms, bandwidth of 31.25 Hz/Px, and echo train length of 2. A central sagittal image of the cervical spine was selected as a locating image for the next T2 FSE transverse-sectional scans at repetition/echo times of 3000/102 ms, Bandwidth of 31.25 Hz/Px, echo train length of 17.

### Histological evaluation

After MRI scanned, the specimens were decalcified firstly, then gradient dehydrated, paraffin-embedded and sectioned with a layer thickness of 5 μm. Hematoxylin-eosin (HE) staining, Safranin O/Fast Green staining, and Alizarin blue staining were performed. The morphology of the specimens was observed under a light microscope after the staining was completed. The extent of cervical IVDs was assessed by the disc degeneration assessment scoring system according to the methods previously reported in the literature [10]. An increase in the score (ranging from 5 to 14) reflected a more severe IVDs degeneration.

### Bacteria detection and quantitative polymerase chain reaction (qPCR) analysis

Cervical disc specimens were collected and genomic DNA was extracted. Immediately, the gene sequencing was carried out after PCR with 16srDNA primers (Table 1), and the corresponding bacterial community was identified by BLAST sequence alignment [11]. The total RNA of cervical disc were extracted separately from the specimens and purified by RNeasy Mini Kit (Qiagen, Valencia, CA, USA). The purity and concentration of the extracted mRNA were determined using a Nano-drop (Biomed, USA) at wavelengths of 260 nm and 280 nm. Reverse transcription was performed at 45°C for 50 minutes and 85°C for 5 minutes using 5× Prime Script RT Master Mix (Cwbio,Taizhou, China). The cDNA was used to perform qPCR for degeneration and inflammation-related genes such as Aggrecan, Col2a1, ADAMTS-5, MMP-3, TNF-α, IL-1β, and the internal reference was β-actin. Reactions using 2 µL cDNA with 10 µL Master Mix (Cwbio, Taizhou, China), 7 µL RNase Free and 2 µL gene-specific forward and reverse PCR primers (synthesized by Sangon biotech Co Ltd, Shanghai, China) (Table 1). PCR reactions were performed at 95°C for 10 mins (activation), followed by 40 cycles of 95°C for 10s, 60°C for 20s, and 72°C for 20s (amplification), and a final extension at 72°C for 1 min, in an AIB Prism 7500 system (Applied Biosystems, Foster City, CA, USA).

**Table 1.** Sequences of primers

Rat Gene	Primer sequences (5’-3’)	
Aggrecan	Forward	GCAGCACAGACACTTCAGGA
	Reverse	CCCACTTTCTACAGGCAAGC
Col2a1	Forward	GGCCAGGATGCCCGAAAATTA
	Reverse	ACCCCTCTCTCCCTTGTCAC
ADAMTS-5	Forward	AGTACAGTTTGCCTACCGCC
	Reverse	GATTTGCCGTTAGGTGGGCA
MMP-3	Forward	TTTGGCCGTCTCTTCCATCC
	Reverse	GCATCGATCTTCTGGACGGT
TNF-α	Forward	CATCCGTTCTCTACCCAGCC
	Reverse	AATTCTGAGCCCGGAGTTGG
IL-1β	Forward	AGGCTGACAGACCCCAAAAG
	Reverse	CTCCACGGGCAAGACATAGG
β-actin	Forward	CTATGAGGGTTACGCGCTCC
	Reverse	ATGTCACGCACGATTTCCCT
P. acnes 16S rDNA	Forward	GGGTTGTAAACCGCTTTCGCCT
	Reverse	GGCACACCCATCTCTGAGCAC
S. epidermidis 16S rDNA	Forward	ATGAAAAAGAGATTTTTATCT
	Reverse	GTTTGGTGACACTCTTAAG

## Statistical analysis

Data was expressed as mean ± standard deviation (STD). All statistical analyses were performed using SPSS 25.0 statistical software (IBM, USA). The differences between groups were calculated using One-way ANOVA and further analyzed by the least significant difference (LSD) method. Ratio differences were calculated using the chi-square test. The *P* ≤ 0.05 shows that the difference is statistically significant (*P < 0.05, **P < 0.01, ***P < 0.005).

## Results

To explore the outcomes of P. acnes and S. epidermidis cervical intervertebral discs (IVDs) infection and the relationship to cervical IVDs degeneration, a model of corresponding bacterial cervical IVDs infection in SD rats was established. The SD rats were randomly divided into five groups: blank group, control group, puncture group, P. acnes group and S. epidermidis group. These rats were assessed for general condition, body weight and MRI scans of the cervical spine before and 2 and 6 weeks after modeling. Subsequently, the blood analysis, histological changes, and expression levels of IVDs degeneration-related mRNA were assayed.

### General condition of animals and changes in body weight

**Figure 4.**
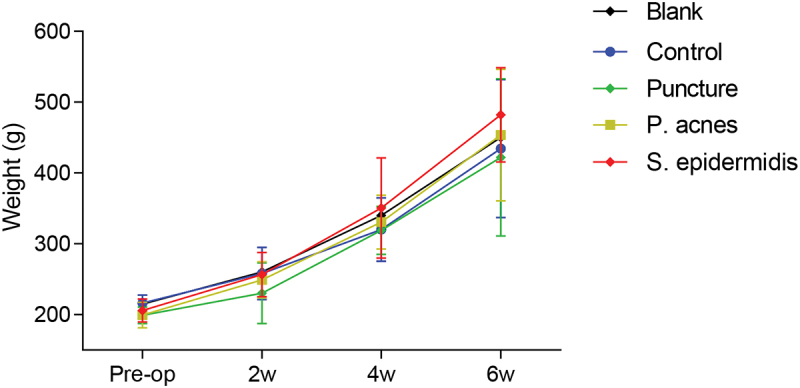
Post-operative weight change in each group of rats.

All animals were successfully operated on for the model, and there were no intraoperative injuries to blood vessels, airway, esophagus, or spinal cord. There were no postoperative complications, and all incisions reached stage I healing around 1 week post operation. There was no accidental death during the perioperative period or the subsequent observation period. The animals gradually increased in weight after surgery and the difference between the groups was not statistically significant (P > 0.05) (Figure 4).

### Blood white blood cell counts (WBC) and percentage of neutrophils (N%)

**Figure 5.**
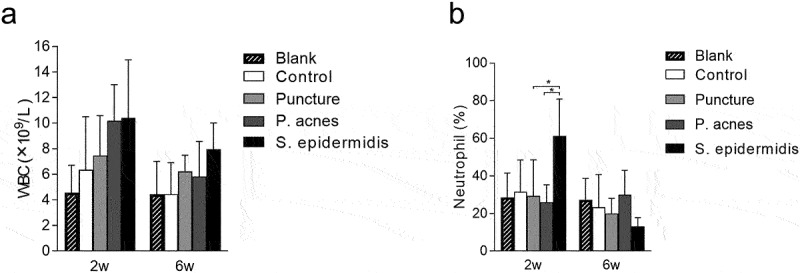
Changes in blood white blood cell (WBC) counts and neutrophils in rats on different time points after operation, (a) white blood cell (WBC) counts, (b) neutrophils percentage.

The WBC counts at 2 weeks postoperatively showed no statistically significant difference between all groups (*P* > 0.05). The WBC in all groups decreased at 6 weeks postoperatively compared to 2 weeks postoperatively, again with no statistically significant difference (*P* > 0.05) (Figure 5a). The percentage of neutrophils was the highest in the S. epidermidis group at 2 weeks postoperatively, with statistically significant differences compared to both the P. acnes group and the puncture group (*P* < 0.05), and non-statistically significant differences compared to the control group (*P* > 0.05). The percentage of neutrophils in the S. epidermidis group decreased at 6 weeks postoperatively, although not statistically different compared with 2 weeks postoperatively. Changes in percentage of neutrophils also did not differ between 2 weeks and 6 weeks postoperatively in other groups (*P* > 0.05) (Figure 5b).

### MRI Imaging

**Figure 6.**
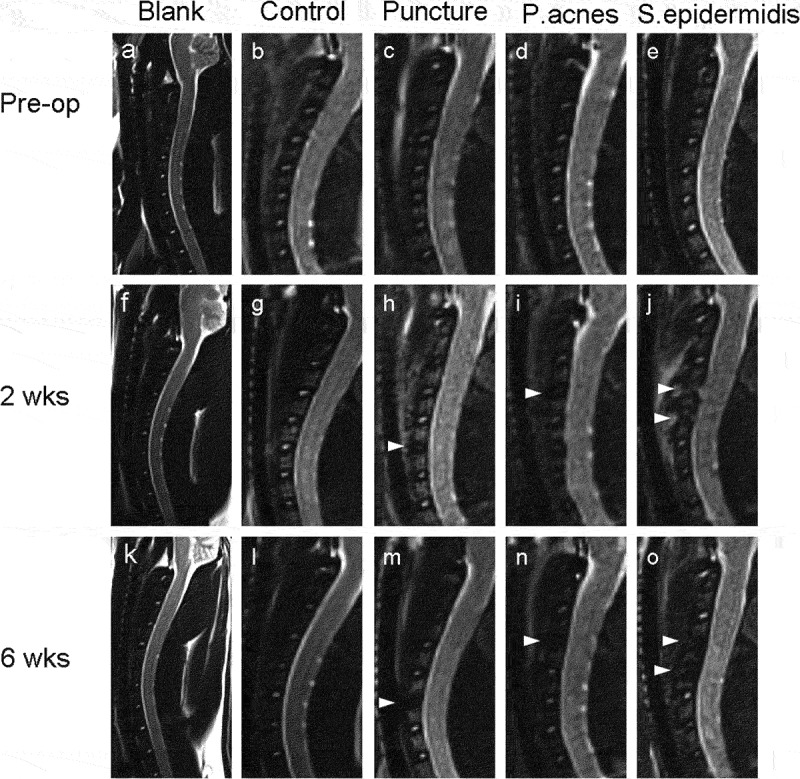
Representative sagittal MRI images of the cervical spine at different time points and for each group of rats. Pre-operative MRI of each group, (a) blank group, (b) control group, (c) puncture group, (d) P. acnes group, (e) S. epidermidis group. The discs marked with arrows are the intervertebral discs of the punctured or injected bacteria. At 2 weeks postoperatively, the (h) puncture group and (i) P. acnes group showed reduced T2 image signal and blurred demarcation between the nucleus pulposus and the annulus fibrosus, whereas the (j) S. epidermidis group showed a more typical manifestation of discitis with inflammatory exudation of the anterior fascia and local abscess formation in the modeled segment. At 6 weeks postoperatively, the (k) blank group, (l)control group showed no change in signal on the T2 image, the (m) puncture group and (n) P. acnes group showed further disc degeneration, with a significant reduction in disc height and a further reduction in the T2 disc signal, resulting in a ‘black disc’; in the (o) S. epidermidis group, the inflammation of the anterior fascial signal subsided, the disc and subchondral bone structure had been destroyed and bone remodeling had occurred.

Preoperative MRI showed high signal in the central part of the target disc on T2 images in all groups, with intact disc structure and normal height. At 2 weeks postoperatively, the puncture group and the P. acnes group showed similar changes in disc and endplate T2-value and disc volume decrease. The blank and control group maintained high T2-value, while the S. epidermidis group had moderate mixed T2 signal (Table 2). The S. epidermidis group showed more typical discitis in the modeled segment with significant loss of disc height, mixed and uneven disc signal, unclear adjacent vertebral bone structure, and extensive high signal in the anterior fascia (Figure 6). At 6 weeks postoperatively, the puncture group and the P. acnes group continued to behave similarly, with further disc degeneration, a marked reduction in disc volume, a further reduction in T2-value, and a poorly defined nucleus pulposus and annulus fibrosus, resulting in a ‘black disc’. In the S. epidermidis group, the anterior fascia signal returned to normal, suggesting that the inflammation had subsided (Figure 6). Meanwhile, the signal of the anterior fascia in the S. epidermidis group returned to normal, while the T2-value and volume of the disc decrease significantly (Table 2). The MRI suggested that the disc and subchondral bone structure had been destroyed and bone remodeling had occurred (Figure 6).

**Table 2.** T2 value, disc volume of cervical disc and endplate region (mean ± SD). Differences were assessed using ANOVA

Group	Pre-op	2wks	6wks	*P-value*
T2 value-disc (ms)				
Blank	61.32 ± 8.35	60.55 ± 7.30	59.33 ± 3.51	0.910
Control	62.55 ± 10.30	58.33 ± 5.61	55.38 ± 6.23	1.012
Puncture	61.82 ± 9.85	48.31 ± 6.35	38.56 ± 5.35	0.04*
P.acnes	61.45 ± 8.95	50.31 ± 5.65	36.56 ± 5.35	0.02*
S. epidermidis	61.76 ± 5.35	65.25 ± 5.82	30.55 ± 4.58	0.005**
*P-value*	0.675	0.05*	0.008**	
T2 value-end plate (ms)				
Blank	56.34 ± 7.48	55.45 ± 6.34	52.54 ± 4.65	0.496
Control	55.34 ± 8.54	51.63 ± 3.25	48.28 ± 5.35	0.245
Puncture	52.74 ± 4.35	45.51 ± 2.38	31.57 ± 3.37	0.043*
P.acnes	54.31 ± 5.25	44.28 ± 4.25	29.28 ± 2.53	0.025*
S. epidermidis	53.56 ± 4.58	55.25 ± 5.54	23.36 ± 3.65	0.001**
*P-value*	0.876	0.03*	0.001**	
Disc volume((mm^3^)				
Blank	5.12 ± 0.41	4.80 ± 1.38	4.50 ± 0.38	0.127
Control	4.92 ± 0.53	4.50 ± 2.30	3.95 ± 2.52	0.145
Puncture	5.23 ± 0.85	3.5 ± 2.42	2.1 ± 1.72	0.042*
P.acnes	5.51 ± 0.67	2.3 ± 0.81	1.8 ± 0.79	0.039*
S. epidermidis	5.31 ± 0.57	1.8 ± 0.87	0.50 ± 0.11	0.007**
*P-value*	0.236	0.045*	0.006**	

*means *P* value<0.05, ** means *P* value<0.01.

### Histology evaluation

**Figure 7.**
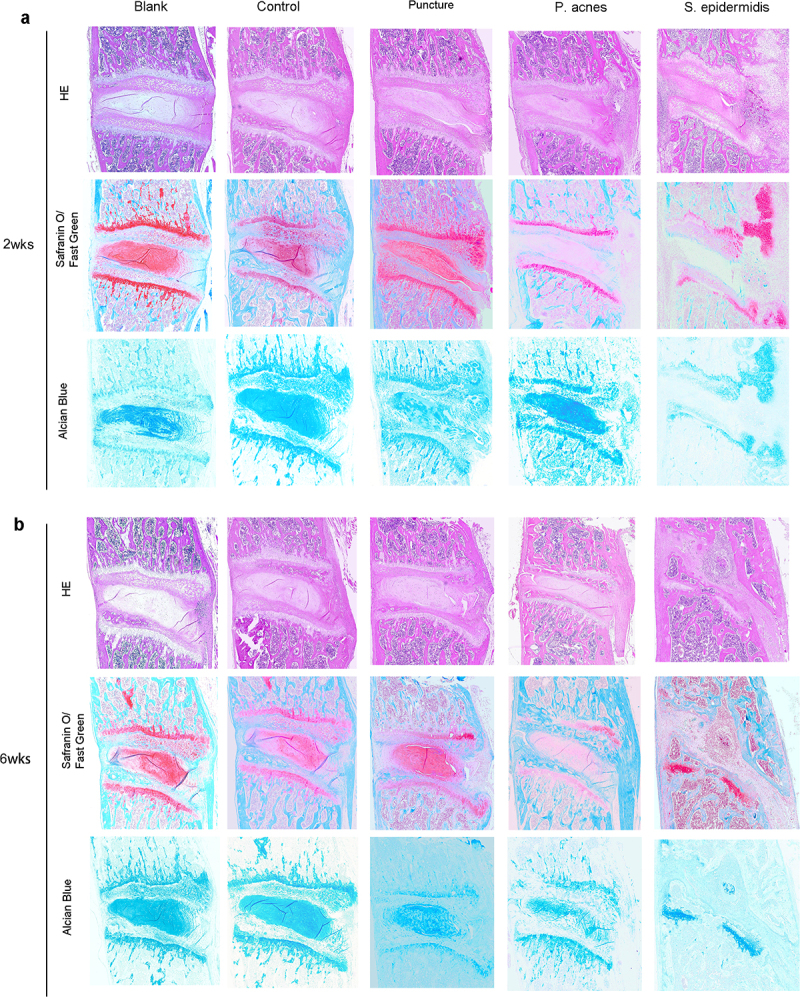
Representative histological images of slices from the control, puncture, P. acnes groups and S. epidermidis group, (a) 2 weeks postoperatively, (b) 6 weeks.

**Figure 8.**
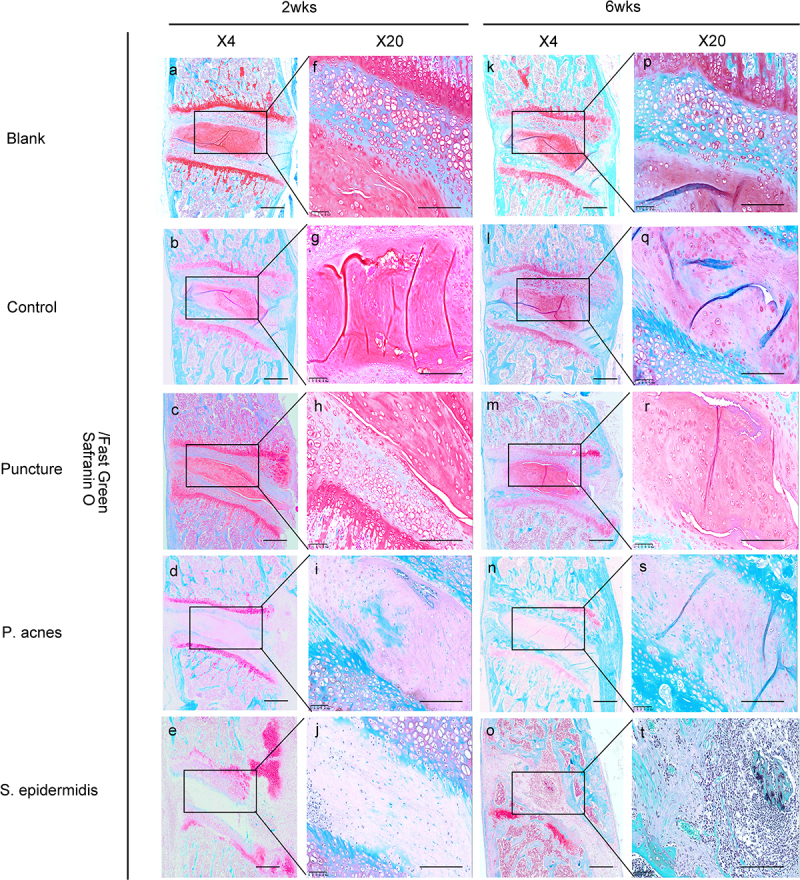
Changes in the bones and cartilage of the cervical vertebrae was shown by Safranin O staining from the blank (a-p), control (b-q), puncture(c-r), P. acnes groups (d-s) and S. epidermidis group(e-t). The rectangular box shows a 40x magnification of the image. Scale bar = 100 μm.

**Figure 9.**
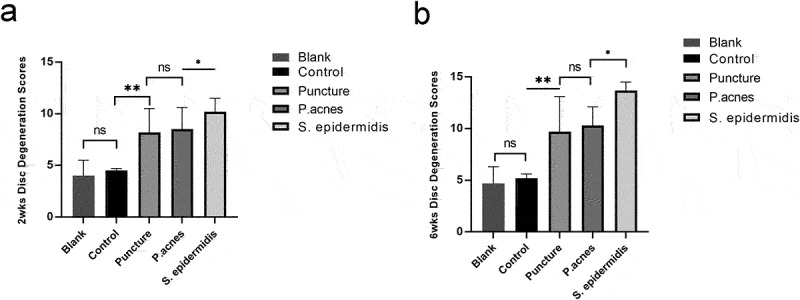
Histological score of modeled segments among the four groups, (a) 2 weeks postoperatively, (b) 6 weeks. *P < 0.05.

The central sagittal HE pathology section of the molded intervertebral disc showed that at 2 weeks postoperatively, the gross and cellular morphology of the blank and control group was basically normal, while cell death in the nucleus pulposus, dehydration of the nucleus pulposus, reduction in disc height and hyperplasia of the fibrous vertebral tissue were seen in the puncture, P. acnes and S. epidermidis groups, with the S. epidermidis group being the most severe, with complete cell death in the central zone of the disc (Figure 7a). In the S. epidermidis group, the cartilage endplate structure was disturbed, with chondrocytes proliferating and endplate bone destruction, whereas in the puncture group and the P. acnes group, the cartilage endplate structure was more intact. (Figure 7a). At 6 weeks postoperatively, the blank and control group showed no significant degeneration of the intervertebral disc or endplate (Figure 7b). In the punctured and P. acnes groups, further matrix degradation occurred at 6 weeks postoperatively (Figure 7b), with ossification of the endplate region and new bone formation at the vertebral margins, with osteosclerosis of the endplate and bone redundancy at the vertebral margins more pronounced in the P. acnes group than in the punctured group, whereas in the S. epidermidis group, bony fusion occurred and the normal disc structure was completely lost due to intervertebral discitis (Figure 8). The histological scores for each group at 2 and 6 weeks are shown in Figure 9 (*P* < 0.05).

### Bacteria detection and quantitative PCR analysis results

**Figure 10.**
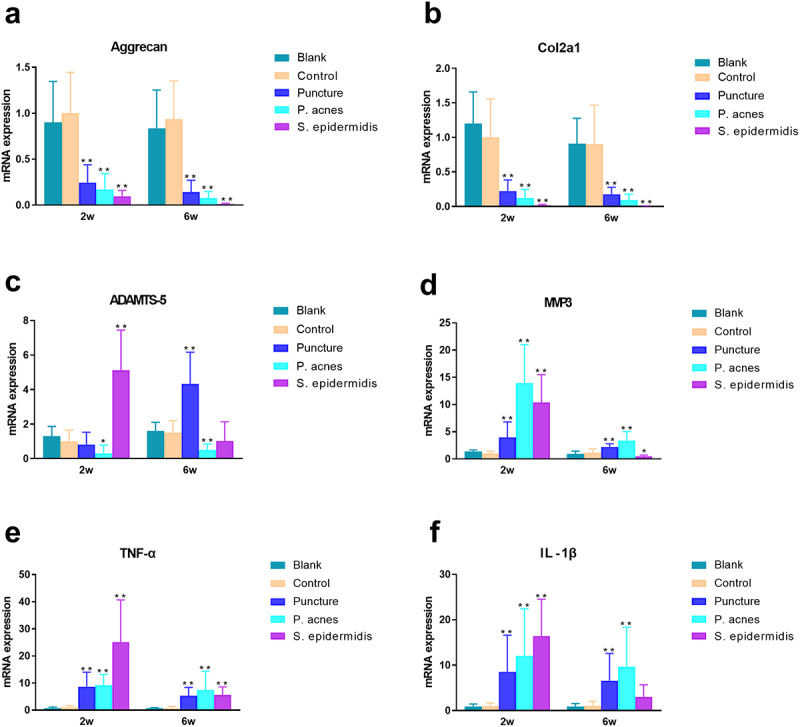
Different time points of quantitative PCR results of modeled intervertebral discs (a) Aggrecan, (b) Col2a1, (c) ADAMTS-5, (d) MMP3, (e) TNF-α, and (f) IL-1β. * P < 0.05, ** P < 0.01.

The results of PCR with 16S rDNA primers showed that the IVD of the molded segments was not contaminated with other bacteria. The results of qPCR for the target genes in the molded segment at 2 and 6 weeks postoperatively are shown in Figure 10. In terms of anabolism, the mRNA expression of Aggrecan and Col2a1 in the puncture, P. acnes and S. epidermidis groups was significantly lower than that in the blank and control group (*P* > 0.05). In terms of metabolism, the expression of ADAMTS-5 gene was only significantly higher in the S. epider- midis group compared to the blank and control group at 2 weeks postoperatively, but not in the puncture and P. acnes groups; while at 6 weeks, the expression of ADAMTS-5 gene was highest in the puncture group and significantly higher than the blank and control group, while the expression in the S. epidermidis group dropped back significantly; the expression of ADAMTS-5 gene in the P. acnes was not upregulated. The expression of MMP-3 gene was highest in the P. acnes group, significantly higher than that in the blank and control group, and higher at 2 weeks than at 6 weeks. Regarding pro-inflammatory factors, the expression of TNF-α and IL-1β genes was significantly higher in the puncture group, P. acnes group and S. epidermidis group than in the blank and control group at 2 weeks postoperatively, with the highest expression in the S. epidermidis group. The expression of TNF-α and IL-1β genes dropped back significantly in the S. epidermidis group puncture group at 6 weeks after surgery, but the expression in the puncture group and P. acnes group was still significantly higher than the blank and control group.

## Discussion

Although the hypothesis that P. acnes infection has a strong clinical association with IVD degeneration has been proven by various studies [3,12,13], these studies mainly concentrated on the lumbar and tail IVDs. Due to the anatomical structures and adjacence to the spinal cord, whether low-virulence infections of cervical IVDs have the same manifestations and reactions is still unknown. Before the IVDs degeneration induced by P. acnes infection was reported, there were many experimental models of IVDs degeneration including mechanical stress model [14], spinal instability model [15], annulus fiber puncture/incision [16], endplate injury [17], nucleus pulposus chemolysis [18], intraperitoneal injection of TNF-α [19], etc. The selection of a well-established animal model as a control for P. acnes infection is crucial for the exploration of the possible mechanism of P. acnes causing IVDs degeneration, which has not been reported in the existing literature. Among these models, annulus fiber puncture is the easiest and most feasible method. Although previous studies have confirmed that puncture with a number 11 blade in lumbar and tail IVDs of rats can induce intervertebral IVDs degeneration successfully [16], our method of puncturing cervical annulus fibrosus with a blade to induce degeneration has not been previously reported. We successfully established a cervical degenerative disc model induced by P. acnes in rats that is very different from discitis induced by S. epidermidis, which can be compared with blade puncture induced IVDs degeneration in the current study.

Findings from the present study verified that cervical IVDs injection of P. acnes could also cause degeneration of the cervical IVDs and adjacent endplate, and this degeneration was very similar to that induced by blade puncture injury of annulus fibrosus. Meanwhile, S. epidermidis inoculation to the cervical IVDs lead to erosion at endplates and subchondral bone structure, and bony fusion in involving cervical IVDs, which are consistent with the manifestation of cervical discitis. These MRI findings corresponded to histological and gene expression data. Whether cervical IVDs were punctured with blade or inoculated with P. acnes or S. epidermidis had little effect on weight gain. The counts of White Blood Cells (WBC) showed no differences at 2 and 6 weeks postoperatively among groups. The highest percentage of neutrophils was found in the S. epidermidis group at 2 weeks postoperatively, and dropped back at 6 weeks postoperatively.

Results of our study showed that the weight of rats were gradually increased postoperatively, and there was no difference in weight gain among groups. Recent changes in weight are an indicator that can reflect current nutritional status [20,21]. Previous studies have shown that there is interaction between inflammation and malnutrition, while severe systemic inflammatory reactions can impair albumin synthesis and lead to malnutrition and weight loss [22]. However, Yang et al [23]. argue that local inflammation response did not affect body weight loss. Therefore, we inferred that both P. acnes and S. epidermidis infected cervical IVDs just produced local inflammatory changes based on the weight change after surgery in the present study. Chen et al [2]. directly inoculated staphylococcus aureus (S. aureus) into the lumbar IVDs of rabbits found the mortality rate can be as high as 67%. As a comparison we injected S. epidermidis to IVDs without finding rats death, the possible reason the dose is low, or the bacteria we inoculated is less virulent than S. aureus. In conclusion, our animal model does not cause a systemic inflammatory response and is a very suitable animal model.

The counts of WBC and neutrophils in the Blood-RT visualized in this study of the P. acnes group were not significantly higher than that of the control group and puncture group, indicating that there was no pyogenic infection reaction. White blood cell and neutrophil counts are frequently elevated after damage and bacterial infection, especially infections by pyogenic bacterial infections [24,25]. This may be due to P. acnes’ lower virulence, combined with the difficulty in surviving in an oxygen-rich environment outside the intervertebral disc. By contrast, the counts of neutrophils were elevated in the S. epidermidis group at 2 weeks postoperatively and dropped back at 6 weeks postoperatively. However, previous studies have shown that WBC and neutrophils counts do not fully reflect the status of the infection, it was also found that the levels of WBC increased, without any bacterial infection [26]. Our findings are consistent with previous studies with pyogenic spondylodiscitis. Thus, P. acnes induced only low-virulence cervical IVDs infections, while S. epidermidis caused pyogenic cervical IVDs infections.

Our results of MRI imaging showed gradually decreased signal intensity on T2WI, and reduced T2 relaxation times and disc volume, since 2 weeks postoperatively in the puncture group and P. acnes group, which indicate cervical IVDs degeneration, consistent with previous studies [27–30]. In contrast, the MRI imaging exhibited mixed hyperintense signals, increased T2 relaxation times, and reduces disc volume in the modeled IVDs and vertebrae on T2WI and erosion at endplates and subchondral bone structure with the abscess surrounding the anterior of IVDs at 2 weeks postoperatively. At 6 weeks postoperatively the MR imaging showed hypointense signals, shorter T2 relaxation times, and bony fusion in involving IVDs in S. epidermidis group, these manifestations are consistent with typical pyogenic discitis. MRI is a powerful method in the diagnosis of IVDs degeneration and spondylodiscitis [31,32]. The accuracy of MRI in the diagnosis of IVDs degeneration reach 100%, and infectious disease with sensitivity was 96%, specificity was 92% and an accuracy of 94% [32,33]. In MRI images, IVDs degeneration is deduced from the finding of decreased signal intensity on T2WI, reduced height, and/or the appearance of fissures in the IVDs [34]. Hong et al [33]. suggested that classic pyogenic discitis will present hyperintense signals related to the IVDs and vertebrae on T2WI and defect or erosion at endplates and vertebral body with/without the abscess enclosing the IVDs.

In this study, based on the HE staining, there were significant changes in cellular morphology, dehydration of the nucleus pulposus, disc height and hyperplasia of the fibrous vertebral tissue of the IVDs from different groups. Differences were also found between the histological images of the P. acnes and S. epidermidis groups. Histological findings also showed that P. acnes induced mild local inflammation of infected IVDs, which agreed with the findings of Białecka et al [35]. S. epidermidis infection of the IVDs could cause a strong local inflammatory response, which was manifested in the early-stage as discitis, destruction of the intervertebral disc and subchondral bone structure, and periosteal reaction in front of the vertebral body [36]. Later, it is manifested by intervertebral disc absorption, vertebral bone repair and remodeling. This pathological appearance is typical for suppurative discitis and is quite different from degenerative changes.

Our quantitative PCR results showed that the relative mRNA expression levels revealed Matrix synthesis in the nucleus pulposus and cartilage was inhibited in the puncture, P. acnes, and S. epider-midis groups. ADAMTS-5 may be the main enzyme involved in matrix breakdown during the acute phase of puncture injury-induced disc degeneration and septic discitis, whereas in the case of P. acnes infection MMP-3 was predominant. These findings in puncture and P. acnes groups were in agreement with published experimental results in IVDs degeneration [37]. However, in S. epidermidis groups the catabolic and anabolic processes is more severe than IVDs degeneration. Inflammatory reactions play a vital role in the progress of IVDs degeneration and discitis [38,39]. Cytokines, such as tumor necrosis factor alpha (TNF-a), interleukin (IL)-1, are crucial factors associated with IVDs degeneration and discitis in human IVDs [39]. The present study likewise clearly demonstrated that the puncture group, P. acnes group and S. epidermidis group all induced a large amount of expression of pro-inflammatory factors (TNF-a, IL-1β) in the IVDs, with the strongest effect of S. epidermidis in the early stage, but its intensity was also short-lived, while the up-regulation of pro-inflammatory factor expression induced by the puncture group and P. acnes group was longer-lasting. Therefore, S. epidermidis is unlikely to be the cause of clinical intervertebral disc degeneration due to its high pathogenicity in IVDs. In the literature, S. epidermidis or CNS detected in intervertebral discs are more likely to be contamination bacteria [5,40].

The high similarity of imaging, bone morphology and histology between the P. acnes group and the puncture group in this study suggests that a considerable part of the clinically observed intervertebral disc degenerative diseases are likely to be induced by P. acnes infection or even P. acnes infection itself. Clinically, the vast majority of patients cannot be diagnosed due to a lack of etiological evidence. Modic changes in endplate on MRI, especially type I Modic changes, are thought to be associated with P. acnes infection [2,3,41,42]. However, in a large proportion of Modic cases, P. acnes is not detected, so Modic change cannot be considered as a direct mark of P. acnes infection [43,44]. There is no specific imaging to diagnose P. acnes infection. Although the findings resemble degenerative changes, the histological morphology of P. acnes infected discs differs slightly in some respects from that of the puncture group. The infiltration of inflammatory cells around the IVDs in the P. acnes group was significantly more than that in the puncture group, and the osteosclerosis of the endplate was more severe, with the local endplate damage, while the endplate in the puncture group remained intact to the end of the experiment. This suggests that although low virulent bacterial infection and mechanical injury have similar manifestations, low virulent bacterial infection tends to lead to a more severe inflammatory response. This may explain why patients with P. acnes infection are clinically found to have more severe pain than patients with simple disc degeneration without P. acnes infection [45].

### Limitation of the study

The limitations of this study are as follows. First, there was no in-depth study on the specific pathogenic mechanisms of P. acnes and S. epidermidis infection, and the pathogenic substances and molecular mechanisms could not be understood so that the huge differences between the two bacteria could not be explained. The second limitation is that this study only evaluated the image, tissue morphology and gene expression of animals, but did not evaluate the animal behavioral scores, so it could not determine whether low virulent bacterial infection could cause nociceptive pain, thus failing to provide experimental evidence for the correlation between P. acnes infection and neck pain. Finally, to investigate the exact quantity of inflammatory indicators, other methods such as enzyme-linked immunosorbent assay (ELISA) would be more opportune. Based on the animal model described in this study, other interventions can be added to influence the process of IVDs degeneration induced by P. acnes in the future, to further understand its internal mechanism and provide a certain reference for clinical treatment.

### Conclusions

P. acnes infection of the cervical IVDs can lead to degenerative changes, whereas S. epidermidis infection leads to the manifestation of septic discitis. The correlation between P. acnes infection and cervical IVDs degeneration found in clinical studies was confirmed.

## Supplementary Material

Supplemental Material
