# Supplementary figures and images for: Different responses of cervical intervertebral disc caused by low and high virulence bacterial infection: a comparative study in rats
Source: Bioengineered. 2022 May 19;13(5):12446–61. doi: 10.1080/21655979.2022.2075305 (PMC9275948; doi:10.1080/21655979.2022.2075305)

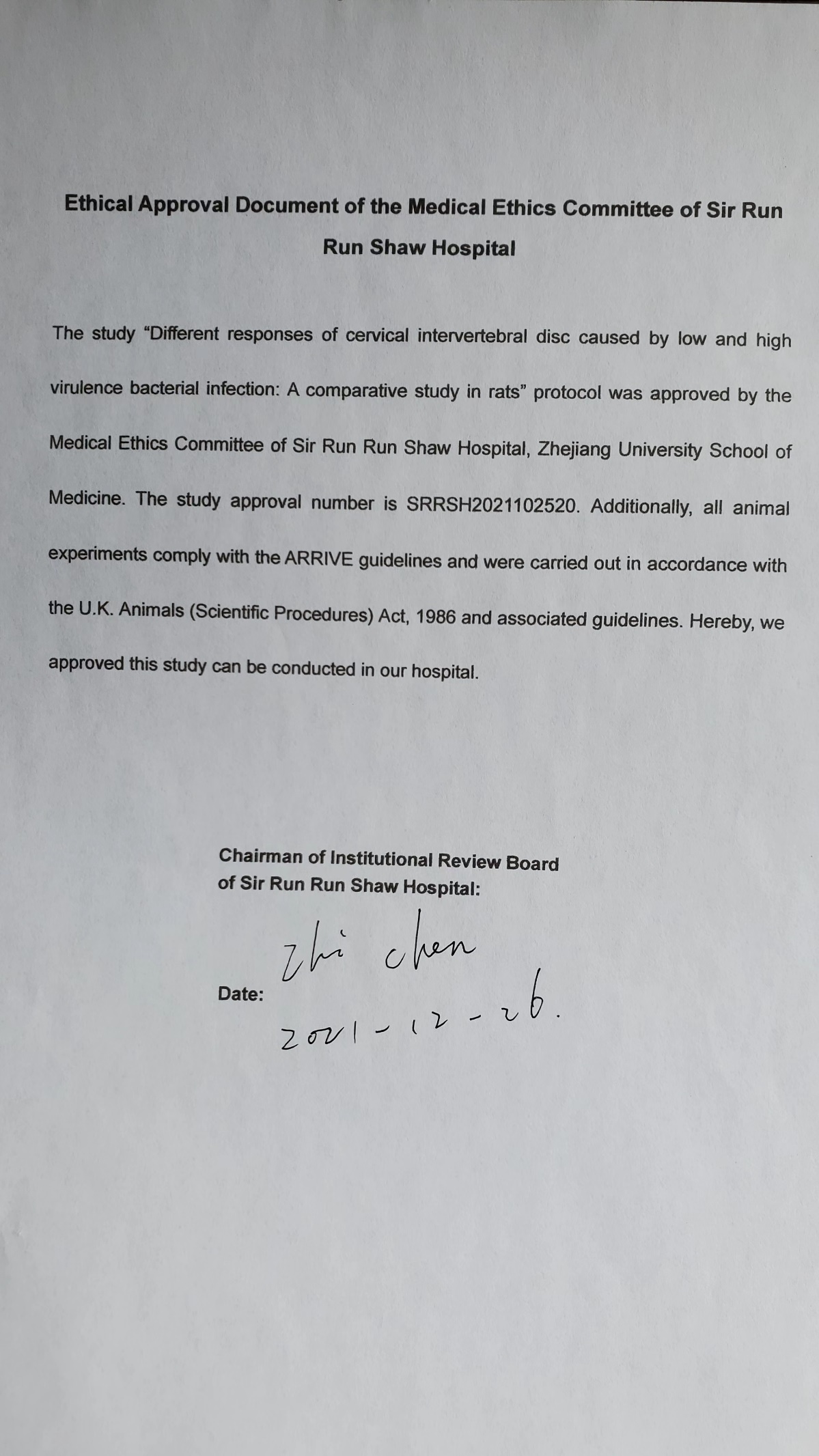

Supplement: Supplemental Material [file KBIE_A_2075305_SM6873.docx]
